# Associations between systemic melatonin and human myopia: A systematic review

**DOI:** 10.1111/opo.13214

**Published:** 2023-08-11

**Authors:** Azfira Hussain, Aparna Gopalakrishnan, Hannah Scott, Chris Seby, Victoria Tang, Lisa Ostrin, Ranjay Chakraborty

**Affiliations:** 1 Myopia Clinic, Unit of Medical Research Foundation, Sankara Nethralaya, Chennai, Tamil Nadu India; 2 Flinders Health and Medical Research Institute: Sleep Health, Flinders University, Adelaide, South Australia Australia; 3 Caring Futures Institute, Myopia and Visual Development Lab, College of Nursing and Health Sciences, Flinders University, Adelaide, South Australia Australia; 4 College of Optometry, University of Houston, Houston, Texas USA

**Keywords:** circadian rhythm, dim light onset melatonin, melatonin, myopia, ocular diurnal rhythm, refractive error

## Abstract

**Purpose:**

Experimental models have implicated the role of melatonin circadian rhythm disruption in refractive error development. Recent studies have examined melatonin concentration and its diurnal patterns on refractive error with equivocal results. This systematic review aimed to summarise the literature on melatonin circadian rhythms in myopia.

**Recent Findings:**

PubMed, EMBASE, Web of Science, Scopus, ProQuest Central, LILACS, Cochrane and Medline databases were searched for papers between January 2010 and December 2022 using defined search terms. Seven studies measured melatonin and circadian rhythms in three biological fluids (blood serum, saliva and urine) in both myopes and non-myopes. Morning melatonin concentrations derived from blood serum varied significantly between studies in individuals aged 10–30 years, with a maximum of 89.45 pg/mL and a minimum of 5.43 pg/mL using liquid chromatography and mass spectrometry. The diurnal variation of salivary melatonin was not significantly different between myopes and emmetropes when measured every 4 h for 24 h and quantified with enzyme-linked immunosorbent assay. Significantly elevated salivary melatonin concentrations were reported in myopes compared with emmetropes, aged 18–30 years when measured hourly from evening until their habitual bedtime using liquid chromatography. However, the relationship between dim light melatonin onset and refractive group was inconsistent between studies. The 6-sulphatoxymelatonin concentration derived from overnight urine volume, measured using a double antibody radioimmunoassay, was found to be significantly lower in myopes (29.17 pg/mL) than emmetropes (42.51 pg/mL).

**Summary:**

The role of melatonin concentration and rhythm in myopia has not been studied extensively. This systematic review confirms conflicting findings across studies, with potential relationships existing. Future studies with uniform methodological approaches are required to ascertain the causal relationship between melatonin dysregulation and myopia in humans.

**Supplementary Information:**

The online version of this article (doi:10.1111/opo.13214) contains supplementary material, which is available to authorized users.

## Key points

- Based on the current literature, there is conflicting evidence for an association between systemic melatonin and myopia in humans.
- Conflicting findings across studies may be due to significant methodological variations in the assessment of melatonin.
- Future studies with larger cohorts, robust methodological approaches and longitudinal design are required to ascertain causal relationships between systemic melatonin and myopia.

## INTRODUCTION

Myopia is a multifactorial condition^1^ that has raised significant global concern in the 21st century. Holden et al.^2^ predicted that half of the world's population would be myopic by 2050. Given the rising prevalence, increased risk of associated ocular pathologies and significant socioeconomic burden, myopia is now identified as one of the immediate concerns by the World Health Organization's global initiative for the elimination of avoidable blindness.^3^ Despite much research, understanding the underlying mechanisms of myopiagenesis and its growing prevalence remains elusive.

Evidence suggests that light exposure and circadian rhythm play a role in refractive development, with numerous studies describing the physiological events associated with circadian modulation of ocular growth.^4–6^ Intrinsic regulators, such as dopamine, melanopsin and melatonin, have been shown to mediate interactions between retinal circuits and signals related to circadian entrainment.^7^ Melatonin, a neuromodulator primarily synthesised by the pineal gland, exhibits diurnal rhythmicity and conveys information about circadian entrainment to the body.^8^ Light exposure mediates systemic melatonin concentration^9–11^; the light activation pathway from intrinsically photosensitive retinal ganglion cells to the pineal gland through the suprachiasmatic nucleus inhibits the systemic release of melatonin.^12^

The gold standard assessment of melatonin circadian timing is the dim light melatonin onset (DLMO), which refers to the endogenous starting point of melatonin secretion collected under dim-light conditions (≤10 lux).^13^ DLMO is the time at which melatonin concentration exceeds an accepted threshold of 10 pM in adults.^14^ In ‘good’ sleepers, DLMO occurs approximately 1–3 h before the average sleep onset.^15^

**FIGURE 1:**
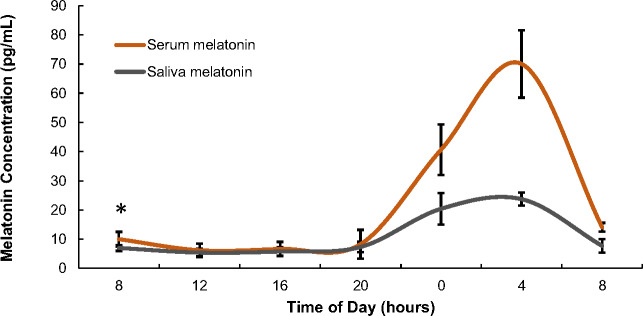
Graphical representation illustrating the changes in melatonin concentration in serum and saliva over a 24-h period in normal healthy adults. The graph was recreated by averaging the mean values of melatonin concentration in serum^17,20,56^ and saliva^17,20,79^ presented in different studies in the age range of 22–53 years. The error bars represent the standard error of the mean. The star indicates that only two studies were available^17,20^ for 08:00 h saliva samples.

A number of factors can influence the DLMO and measured concentration of melatonin, including age,^16^ time of day^17^ and methods of analysis of melatonin assays.^18^ Diurnal variation in melatonin concentration in two commonly measured body fluids, serum and saliva, over a 24-h period is shown in Figure 1. Serum melatonin, which requires venipuncture or in-dwelling cannulas for blood collection, is considered the gold standard for quantification. However, salivary assessment of melatonin is more common and widely accepted in practice due to its easy and noninvasive nature.^19^ The concentration of serum melatonin in young and healthy adults can vary from approximately 5 to 100 pg/mL, depending on the time of day. Serum melatonin concentration is highest at night between 02:00 and 04:00 h (60–80 pg/mL) and lowest during the day (typically ≤10 pg/mL). During the day, salivary and serum melatonin concentrations are generally similar (≤10 pg/mL), whereas at night, during the peak melatonin secretin phase, saliva melatonin can be considerably lower than the serum melatonin concentration (Figure 1). At the peak, salivary melatonin levels are typically around 30%–40% of the corresponding serum levels.^20,21^ Despite these differences, the peak timing of melatonin secretion and melatonin concentration during the rising and descending phases of secretion are strongly correlated between saliva and serum samples.^20^ DLMO is typically earliest in children below 10 years of age and latest in late adolescence (around 20 years of age), and thereafter slowly advances with age in older individuals. Additionally, the peak serum melatonin concentration deceases from approximately 80 pg/mL in 10- to 19-year-olds to 64 pg/mL in 40- to 49-year-olds, and further declines to 25 pg/mL in 80- to 89-year-olds, with considerable inter-individual variability.^22^

Accumulating evidence provides support for a role of light exposure in myopia.^11,23–25^ Increased time outdoors in children is associated with a decrease in myopia onset, with some studies also reporting decreased myopic progression over time.^26^ Based on these findings, speculation exists as to whether there are associations between melatonin, light exposure and myopia development. However, little is known about the pathophysiological association between melatonin and myopia. Recent studies have investigated systemic melatonin concentrations in non-myopic and myopic individuals. However, findings have been conflicting and conclusions have been ambiguous.^27–33^ Furthermore, potential mechanisms behind the relationship have not been elucidated.

To date, data published in this area are heterogeneous. Therefore, this review aimed to summarise and critically review myopia-related systemic melatonin concentrations and to examine associations between melatonin and the refractive state based on the existing literature. These assimilated findings may contribute to a better understanding of the role of melatonin in myopia.

## METHODOLOGY

### Study focus and eligibility criteria

This systematic review was conducted in accordance with PRISMA (Preferred Reporting Items for Systematic Review and Meta-analyses) guidelines^34^ and was registered with the International Prospective Register of Systematic Reviews (PROSPERO) (ID number CRD42022368436). All studies that measured the concentration of serum melatonin in any specimen, including blood, saliva or urine in participants with myopia, and compared these concentrations against emmetropic or non-myopic participants were included in this systematic review.

Studies were excluded based on the following criteria: (1) did not compare melatonin concentration between myopes and non-myopes; (2) only used animal models or in vitro conditions; (3) having an unmatched design, such as case reports and reviews; (4) involving conditions other than myopia and (5) included participants on myopia control interventions.

Myopia was defined as a spherical equivalent refractive error of ≤−0.50 D based on the International Myopia Institute classification.^35^ All eligible studies reported melatonin concentration in pg/mL.

### Search strategy

**FIGURE 2:**
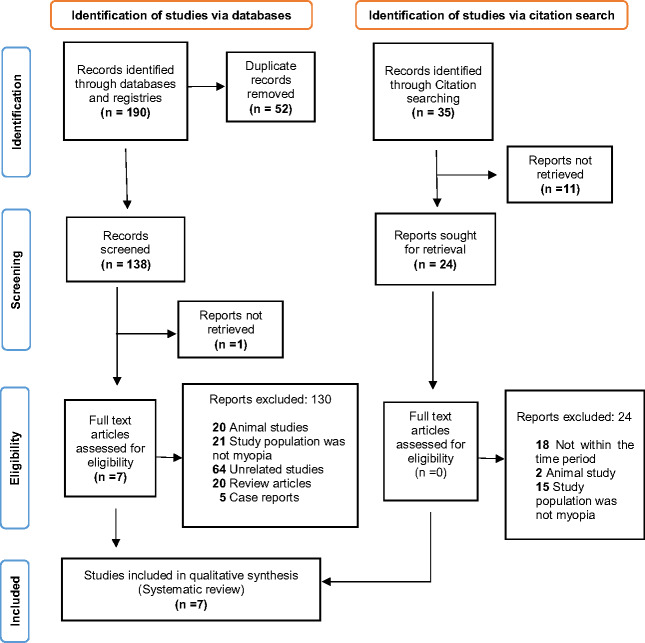
Preferred Reporting Items for Systematic Review and Meta-analyses flow diagram of literature search and study selection.

The search protocol is shown in Figure 2. Original peer-reviewed (observational) studies from PubMed, EMBASE, Web of Science, Scopus, ProQuest central, LILACS, Cochrane and Medline (Ovid) databases were searched. The following title/abstract search terms [“Myopia” OR “Myopes” OR “Myopic” OR “Near-sightedness” OR “Short-sightedness”] AND [“Melatonin” OR “Melatonin circadian rhythm” OR “Melatonin rhythm” OR “Melatonin receptors”] were applied. Publication dates were constrained to 1 January 2010 and 31 December 2022. Only English language full-text articles were included. The search terms for each database are provided in Appendix S1. Articles identified from the databases were imported to Mendeley software (version 1.19.4, mendeley.com) and duplicate articles were removed manually. Additionally, manual scanning of the reference lists of all eligible studies was performed to obtain potential studies of interest.

### Study selection and data extraction

The titles and abstracts of the articles identified by database and citation search were independently screened by two reviewers (AH and AG) based on the eligibility criteria for full-text review. Conflicts over inclusion were adjudicated by the third reviewer (RC). The extracted studies were compared by two reviewers, and inconsistencies were resolved by consensus. Both reviewers extracted information from retrieved studies using an extraction template. The following aspects were taken into consideration: the first author's name, publication year, study title, study design, study participants' age and baseline risk factors, outcome measurement and assessment, reported melatonin concentration, statistical analysis of results and limitations highlighted by the study investigators.

### Quality assessment

All retrieved studies were appraised independently by the reviewers following data extraction. The Newcastle–Ottawa Quality Assessment Scale (NOS; adapted for non-randomised observational studies)^36^ was used to assess the methodological quality and risk of bias of the included studies. The adapted version of the NOS for cross-sectional and cohort studies by Herzog et al.^37^ was applied for the purpose of this study. The NOS tool is a star rating system that allocates a maximum of nine stars based on the description of methods and the level of detail provided in the studies included. It was used to grade the studies across three domains: (a) selection (5 points), (b) comparability (1 point) and (c) outcome of interest (3 points). The selection domain was further subdivided into: (a) representativeness of samples pertaining to how well the myopic and emmetropic participants represented the ‘experimental’ and ‘control’ populations; (b) information on sample size; (c) non-response rate pertaining to a clear description of when and why a melatonin sample was not collected from a participant and (d) ascertaining exposures that were used to determine and define myopia (such as cycloplegic refraction). The second domain of compatibility rated whether the study groups were matched in terms of the participant's demographics such as age and gender. The third domain, outcome of interest, evaluated the appropriateness of the research methods and the statistical tests used for the evaluation of study outcomes.

## RESULTS

### Study characteristics

An initial search identified 190 records from electronic databases and 35 from references of the selected studies, of which 52 were duplicates. The abstracts of 138 records were screened by two reviewers independently, excluding one record that did not meet the selection criteria. The remaining 137 records underwent a full review. Overall, seven studies fulfilled the inclusion criteria (Table 1). All seven studies were cross-sectional and described associations between melatonin concentration and myopia. The sample size ranged from 18 to 120 participants and participant ages ranged from 5 to 41 years.

**TABLE 1:** Details of studies included in the systematic review.

References	Diagnostic criteria	Age (years)	Population	Bio fluid measured	Technique
Kearney et al.^27^	Myopia (SER ≤ −0.50 D) Emmetropia (SER > +0.50 D)	18–20	Phase 1 25 Myopes 29 Non-myopes Phase 2 22 Myopes 23 Non-myopes	Serum (melatonin)	SPE-MS/MS
Abbott et al.^29^	Myopia (SER < −0.50 D) Emmetropia (SER +1.25 to −0.50 D)	17–40	31 Myopes 19 Emmetropes	Saliva (melatonin)	ELISA
Burfield et al.^28^	Myopia (SER ≤ −0.75 D) Emmetropia (SER +1.50 to >−0.75 D)	22–41	25 Myopes 17 Emmetropes	Saliva (melatonin)	ELISA
Ostrin et al.^33^	No definition provided	5–14	6 Myopes 12 Emmetropes	Saliva (melatonin)	ELISA
Flanagan et al.^30^	Myopia (SER ≤ −0.50 D) Emmetropia (SER > +0.50 D)	18–30	28 Myopes 23 Emmetropes	Serum (melatonin) & saliva (DLMO & melatonin)	SPE with HPLC-MS/MS
Chakraborty et al.^31^	Myopia (SER < −1.50 D) Emmetropia (SER ± 0.50 D)	18–25	18 Myopes 14 Emmetropes	Urine (aMT6) & saliva (DLMO)	Double antibody RIA
Kumar et al.^32^	Myopia (SER < −1.00 D) Emmetropia (SER ± 0.25 D)	10–25	60 Myopes 60 Emmetropes	Serum (melatonin)	HPLC-MS

Abbreviations: aMT6, 6-sulfatoxymelatonin; D, dioptre; DLMO, dim light melatonin onset; ELISA, enzyme-linked immunosorbent assay; RIA, radioimmunoassay; SER, spherical equivalent refraction; SPE MS/MS, solid phase extraction mass spectrometry; SPE with HPLC-MS, solid phase extraction with high performance liquid chromatography-mass spectrometry.

Based on the source of the biological samples analysed, the studies were categorised into melatonin measured in blood serum, saliva and urine. Two studies examined melatonin concentration in blood serum,^27,32^ three examined saliva samples^28,29,33^ and two examined two types of biological fluids each (saliva and urine,^31^ and saliva and serum^30^). In terms of analytical platforms, three studies employed liquid chromatography-mass spectrometry and tandem (LC–MS and LC–MS/MS),^27,30,32^ three used enzyme-linked immunosorbent assay (ELISA)^28,29,33^ and one study used double antibody radioimmunoassay (RIA).^31^

### Quality assessment

The quality of the studies was assessed with the NOS tool. The score summary of each study is presented in Table 2. The average quality assessment score ranged from 4 to 7. With the exception of one study, all described the selection strategy for myopic and emmetropic participants. Sample size justification was provided by only one study. In addition, the non-response rate was described by only four studies. Only four studies presented the statistical comparability of age between the two refractive groups, and only two studies defined myopia based on cycloplegic refraction. However, all studies clearly described the assessment of their outcome variables and the statistical tool used for analysis, and no concerns were noted regarding these two biases.

**TABLE 2:** Quality assessment of studies using Newcastle-Ottawa Scale tool.

References	Selection				Comparability	Outcome		Overall score
	Representative of sample	Sample size	Non-response rate	Ascertainment of exposure		Assessment of outcome	Statistical tool	
Kearney et al.^27^	*^a^	*	*			*	*	5
Abbott et al.^29^				*	*	*	*	4
Burfield et al.^28^	*			*		*	*	4
Ostrin et al.^33^	*		*			*	*	4
Flanagan et al.^30^	*				*	*	*	4
Kumar et al.^32^	*	*	*	*	*	*	*	7
Chakraborty et al.^31^	*		*		*	*	*	5

^a^ A maximum of one star was awarded for each numbered item in the selection and outcome categories.

### Serum melatonin and myopia

The main outcomes of the included studies are summarised in Table 3. The median serum melatonin concentration derived from blood collected at a single time point in the morning (between 08:30 and 10:00 h) varied significantly between studies, ranging from a minimum of 5.43 pg/mL to a maximum of 89.45 pg/mL when analysed using MS.^27,30,32^ Serum melatonin concentration was found to be significantly higher in myopes than in emmetropes across the age range of 10–30 years.^27,30,32^ There was no seasonal variation in morning melatonin concentration, measured in the same participants, across two seasons (summer and winter).^27^

**TABLE 3:** Summary of the main outcome of the included studies for melatonin concentration and timings (continued overleaf).

Bio fluid	References	Refractive errors (D)	Age range (years)	Melatonin concentration (pg/mL) and timings (h:min)		Sampling time	Technique	Key findings
				Myopes	Emmetropes			
Serum	Flanagan et al.^30^	Myopia (SER ≤ −0.50) Emmetropia (SER > +0.50)	18–30	Median (IQR) 5.43 (4.32–6.53)	Median (IQR) 3.45 (2.75–4.52)	Single time point 08:30–09:00 h	SPE with HPLC-MS/MS	Significantly higher melatonin concentration among myopes than emmetropes
	Kearney et al.^27^	Myopia (SER ≤ −0.50)	18–20	Phase 1 (Summer)		Single time point 08:30–10:00 h	SPE-MS/MS	Significantly higher melatonin concentration among myopes than controls in both seasons
				Median (IQR) 15.5 (11.9–19.9)	Median (IQR) 5.5 (3.7–7.4)			
				Emmetropia (SER > +0.50)	Phase 2 (Winter)			
				Median (IQR) 14.2 (10.1–16.6)	Median (IQR) 6.9 (4.1–9.1)			
	Kumar et al.^32^	Myopia (SER < −1.00) Emmetropia (SER ± 0.25)	10–25	Median (IQR) 89.45 (7.46–141.81)	Median (IQR) 52.83 (21.18–259.95)	Single time point 08:00–09:00 h	HPLC-MS	Significantly higher melatonin concentration among myopes than emmetropes
Saliva								
One time point	Abbott et al.^29^	Myopia (SER <0.50) Emmetropia (SER +1.25 to −0.50)	17–40	Mean ± SD 7.1 ± 4.0	Mean ± SD 6.5 ± 2.5	Single time point 09:00–11:00 h	ELISA	1. No significant difference between myopes and emmetropes; 2. Higher morning melatonin is associated with red and blue light exposure for 7 days
Diurnal variation and acrophase	Burfield et al.^28^	Myopia (SER ≤ −0.75) Emmetropia (SER +1.50 to > −0.75)	22–41	Mean ± SD 12.41 ± 1.0	Mean ± SD 10.64 ± 1.22	Every 4 h for 24 h (08:00 h onwards)	ELISA	1. No significant difference between myopes and emmetropes in mean daily melatonin; 2. Low concentration during the lights on period (12:00 h), and high during the lights off period (04:00 h)
				Mean ± SD Amplitude of melatonin diurnal variation 26.95 ± 1.88				
				Acrophase: 03:11 h				
	Ostrin et al.^33^	No definition provided	5–14	Mean ± SD Amplitude of melatonin diurnal variation 29.40 ± 3.15		Every 4 h for 24 h (08:00 h onwards)	ELISA	Low concentration during the lights on period (16:00 h), and high during the lights off period (04:00 h)
				Acrophase: 02:22 h				
	Flanagan et al.^30^	Myopia (SER ≤ −0.50) Emmetropia (SER < +2.00)	18–30	Median (IQR) 08:00 3.30 (2.37–4.34) 00:00 35.35 (30.71–37.44)	Median (IQR) 08:00 1.48 (0.86–2.53) 00:00 27.22 (24.16–29.13)	Every hour from 19:00 h until habitual bedtime and a single measure at 08:00 h	SPE with HPLC-MS/MS	1. Significantly higher melatonin concentration among myopes than emmetropes at different time points; 2. Lowest concentration during morning (08:00 h), and highest during the night (00:00 h)
DLMO	Chakraborty et al.^31^	Myopia (SER < −1.50) Emmetropia (SER ± 0.50)	18–25	Mean ± SD (h:min) 10.31 ± 1.76 (22:19)	Mean ± SD (h:min) 9.12 ± 1.43 (21:07)	Every half hour, starting 5 h prior and continuing to 2 h after habitual bedtime	Double antibody RIA	Significant phase delay of 1 h 12 min in myopes compared to emmetropes
	Flanagan et al.^30^	Myopia (SER ≤ −0.50) Emmetropia (SER < +2.00)	18–30	Median (IQR) (h:min:s) 20:22:49 (20:16:00–20:32:59)	Median (IQR) (h:min:s) 20:22:43 (20:18:18–20:28:22)	Every hour from 19:00 h until habitual bedtime	SPE with HPLC-MS/MS	No significant difference between myopes and emmetropes
Urine	Chakraborty et al.^31^	Myopia (SER < −1.50) Emmetropia (SER ± 0.50)	18–25	Mean ± SD 29.17 ± 18.67	Mean ± SD 42.51 ± 23.97	Overnight urine void (18:00 h to 08:00 h)	Double antibody RIA	Significantly higher melatonin concentration among emmetropes than myopes

*Note*: Values reported for melatonin concentrations are given in pg/mL and DLMO values are given in h:min. Age range is in years.

Abbreviations: aMT6, 6-sulfatoxymelatonin; D, dioptre; DLMO, dim light melatonin onset; ELISA, enzyme-linked immunosorbent assay; IQR, interquartile range; RIA, radioimmunoassay; SD, standard deviation; SER, spherical equivalent refraction; SPE MS/MS, solid phase extraction with mass spectrometry; SPE with HPLC-MS/MS, solid phase extraction with high performance liquid chromatography-mass spectrometry.

### Salivary melatonin and myopia

Four studies assessed differences in melatonin concentration^29^ and melatonin circadian rhythms or timing^28,30,33^ in myopes and emmetropes using saliva samples.

### Salivary melatonin measured at one time point

Regarding melatonin concentration, Abbott et al.^29^ found no significant differences between refractive groups when quantified at a single time point (09:00–11:00 h) using ELISA in adults aged 17–40 years. On the contrary, Flanagan et al.^30^ found that morning salivary melatonin concentrations, measured between 08:30–10:00 h, were significantly higher in myopes (3.37 pg/mL) than in emmetropes (1.54 pg/mL) when analysed using mass spectrometry (MS) in adults aged 18–30 years.

### Salivary melatonin for the assessment of diurnal variations

Studies evaluating the diurnal pattern of salivary melatonin concentration all showed a sharp rise at night and a decline during the day.^28,30,33^ When measured every 4 h over a 24-h period, Burfield et al.^28^ found no significant difference in the acrophase of melatonin concentration (03:11 h) between adult myopes and emmetropes (ages 22–41 years). Similarly, Ostrin et al.^33^ evaluated diurnal variations of systemic melatonin rhythms in children aged 5–14 years, and found the nocturnal melatonin acrophase to occur at 02:22 h. However, the study did not assess differences between refractive error groups.

### Salivary melatonin for the DLMO assessment

Flanagan et al.^30^ showed significantly elevated salivary melatonin concentrations in myopes compared to emmetropes (ages 18–30 years), when measured hourly from 19:00 to 23:00 h at ≤30 lux using the LC–MS/MS technique. Interestingly, there was no phase difference in melatonin rhythm timing (DLMO) between the two refractive groups despite significantly higher salivary melatonin concentrations in myopes in the evening. A recent study by Chakraborty et al. found a DLMO phase-delay of 1 h 12 min in young adult myopes compared with emmetropes (ages 18–25 years) when saliva samples were collected every half hour for 7 h in a sleep lab (<10 lux lighting) and analysed using double antibody RIA.^31^

### Urinary melatonin and myopia

Chakraborty et al.^31^ also assessed total melatonin production via urinary 6-sulphatoxymelatonin (aMT6s, a metabolite of melatonin) concentration from urine voids collected overnight and observed a significantly lower level of aMT6s in young adult myopes compared to emmetropes.

In summary, three of the seven studies found a significantly higher concentration of serum melatonin in young adult myopes than emmetropes in the morning. However, this association between refractive error and morning melatonin concentration was found to be inconsistent with salivary melatonin. There were no differences in the diurnal variations of melatonin concentration between adult myopes and emmetropes. Salivary DLMO results were conflicting, as one study reported a significant phase delay in the melatonin circadian timing of myopes compared to emmetropes, while another investigation found no difference in the DLMO timing between the two groups. Finally, one study found overnight urinary melatonin output to be significantly lower in myopes than in emmetropes.

## DISCUSSION

This review aimed to summarise the relevant existing literature on melatonin concentration and diurnal patterns in myopia. Across studies, there was conflicting evidence for an association between systemic melatonin and myopia. The disparate findings between studies, at least in part, are likely related to variations in study design regarding the included population and methods of analysis.

There has been a growing interest in understanding the role of circadian rhythm in myopia development,^38^ based on a large body of evidence in both animal models and humans that light exposure, the most important Zeitgeber for circadian entrainment, is involved in eye growth. Studies in animals show that disruption of daily light:dark cycles alter normal eye growth and lead to refractive errors.^39–41^ In mice, retinal-specific knockouts of the clock gene Bmal1 (*Bmal1*^*−/−*^) and the melanopsin gene Opn4 (*Opn4*^*−/−*^) were shown to be associated with abnormal refractive development and myopia.^42,43^ In addition, investigations examining gene expression in animal models of myopia have reported changes in expression of mRNAs associated with circadian clock genes,^44^ while genome wide association studies have reported single nucleotide polymorphisms in similar genes associated with myopia.^45^

Several recent studies in children and young adults with myopia have reported delayed sleep, shorter sleep duration and poor sleep quality compared to emmetropes.^31,46–49^ Since melatonin is the physiological regulator of sleep,^8^ these findings suggest that melatonin and/or circadian rhythm dysregulation may be linked to myopia. However, these findings have been contradicted by other investigations.^50,51^

All three studies that examined morning melatonin levels in blood serum found significantly higher morning melatonin concentrations in myopes compared with non-myopes. Given that higher concentrations of serum melatonin are correlated with higher degrees of myopia,^27^ the inclusion of individuals with high myopia (up to −20.75 D) by Kumar et al.^32^ may have contributed to the higher mean melatonin concentrations. Across the studies included here, there was a wide range of reported morning blood serum melatonin concentrations. For example, the morning melatonin concentration reported by Kumar et al.^32^ was approximately 6- to 8-fold higher than the observations of Flanagan et al.^30^ and Kearny et al.^27^ Kumar et al. included a younger age range (10–25 years) than the other two studies (19–24 years). Previous investigations have found that adolescents (during puberty) have higher melatonin concentrations than young adults,^52,53^ which could also have accounted for the higher melatonin concentrations.

Two studies included in this review used saliva samples collected at a single time point (between 08:30 and 11:00 h) to examine the differences in melatonin concentration between myopes and emmetropes.^29,30^ Whilst Abbott et al.^29^ found no significant difference in salivary melatonin concentrations between refractive groups, Flanagan et al. found morning melatonin concentrations to be significantly elevated in myopes compared to emmetropes. These disparities in outcomes between studies may reflect differences in melatonin quantification methods (ELISA vs. LC–MS). Based on a review of 84 published papers, Kennaway et al. reported that several ELISA kits were poorly validated and could grossly overestimate the morning melatonin levels by up to 10–40 times in comparison with RIA and MS, respectively.^54^ Whilst these variations could certainly influence the overall results, how exactly they might impact the measured melatonin concentrations in myopes versus emmetropes remains unknown. Importantly, as melatonin synthesis and release are strongly light dependent and circadian, and there are large inter-individual differences in circulating levels, reporting only a single time point may be difficult to interpret.^55^

Consistent with the literature,^56^ significant diurnal variation of melatonin concentration was observed in children^33^ and young adults^28^; melatonin concentration was significantly higher at night and lower during the day. Burfield et al.^28^ found no differences in the peak melatonin circadian timing (occurring at 03:11 h) between adult myopes and non-myopes. In these studies, melatonin concentration was assessed every 4 h.

Other studies aimed to determine the DLMO for each participant and compare them across refractive error groups.^30,31^ Flanagan et al.^30^ found no significant differences in DLMO between refractive error groups, while Chakraborty et al.^31^ reported a significant DLMO phase-delay of 1 h 12 min in myopes (22:19 h) compared with emmetropes (21:07 h). Differing findings between studies may have been due to differing methodological approaches. Flanagan et al. collected hourly saliva samples from 19:00 h until each participant's habitual bedtime while in the laboratory with ambient illumination of less than 30 lux. Where habitual bedtime had not been reached after the 23:00 h sample, participants were allowed to return home and requested to continue hourly sampling at home in a similar dim environment until habitual bedtime. This presents inadvertent limitations: (a) the amount of light exposure outside the lab environment after 23:00 h could not be monitored or controlled^57^; (b) the hourly sampling of saliva may have been insufficient to capture small changes in melatonin rhythm timing^58^ and (c) DLMO was measured under ≤30 lux illumination compared to the recommended ≤10 lux lighting.^59^ On the other hand, in the latter study, saliva samples were collected every half hour for 7 h, beginning 5 h before and ending 2 h after each participant's average sleep onset. Samples were collected in a sleep laboratory under <10 lux to capture the complete DLMO profile. Additionally, the two studies used different methods of melatonin analysis (LC–MS/MS by Flanagan et al.^30^ vs. double antibody RIA by Chakraborty et al.^31^). Saliva RIA has a very high sensitivity of 1 pg/mL and is able to detect and quantify low concentrations of melatonin in saliva samples.^18^ Mass spectrometry is also highly sensitive and offers a comparable range of detection and quantification of melatonin in saliva.^60^ Despite comparable sensitivities, the nocturnal melatonin levels measured by RIA (range approximately 40–80 pg/mL) can be slightly higher than MS (approximately 30–50 pg/mL) due to procedural differences in the processing of the samples. These variations could potentially affect the overall melatonin concentration measured in these studies.

Melatonin concentration in urine is typically measured using aMT6s, the primary metabolite in urine.^61^ Studies have shown a high degree of correlation in the peak timing of melatonin release between measurements of urinary aMT6s and plasma and serum melatonin.^62,63^ Chakraborty et al.^31^ found that overnight melatonin secretion or output, as measured by urinary aMT6s concentration, was significantly lower in myopes than in emmetropes, and that there was a linear reduction in melatonin output with increasing severity of myopia. The urinary aMT6s concentration represented the total melatonin produced overnight (i.e., the melatonin secreted between 18:00 and 08:00 h the following morning). These findings were in contrast with other studies that either found no significant differences in melatonin concentration between refractive groups^29^ or significantly higher melatonin concentration in myopes.^27,30,32^ As discussed previously, these studies analysed serum melatonin concentration based on a single saliva or blood sample collected in the morning^27,30,32^ or with five or six saliva samples collected hourly in the evening,^30^ none of which reflects the total circulating melatonin in the system. Since Chakraborty et al.^31^ measured the total cumulative circulating melatonin overnight in urine (as opposed to a single time point in saliva or serum), the melatonin output findings from their study cannot be directly compared with other studies. While urinary aMT6S provides a more comprehensive analysis of the total overnight melatonin production, the measure may be affected by renal secretions, especially creatinine.^64^ Nevertheless, the accuracy and reliability of urinary aMT6s make it an excellent candidate for future myopia research.^61^

The ‘absolute’ concentration of melatonin can vary significantly depending on the time of day and the biological fluid (serum, saliva or melatonin) from which it is collected. Therefore, in this review, we only compared those studies conducted at the same time of day and/or using the same biological fluids. To avoid any confusion in the interpretation of results with regard to time of day, comparisons of melatonin concentration in myopes and emmetropes were made separately for studies in which melatonin was measured: (a) at one time point in the morning; (b) multiple times during the day for diurnal assessment and (c) for DLMO assessment for individual biological fluids (Table 3). At any given time of day, there are significant differences in the absolute concentration of melatonin in saliva, serum and urine.^17^ As shown in Figure 1, salivary melatonin concentration is 30%–40% of the corresponding serum concentration. Melatonin in blood is bound to albumin and alpha-1-acid glycoprotein, whereas melatonin in saliva represents the free, unbound plasma melatonin levels.^65,66^ Furthermore, salivary and serum melatonin values cannot be compared with urinary melatonin levels. This is because the melatonin found in urine is its metabolite, aMT6s, and not a direct measure of melatonin in plasma.^17,63^ Importantly, the melatonin output is calculated based on the amount of the melatonin metabolite in urine collected across several hours (2–4 h or more), which is then multiplied by the total volume of urine. For urinary aMT6S, the total amount of melatonin excreted over time is reported, rather than melatonin concentration at a specific time point, as is the case for saliva or serum. The concentration of melatonin in urine is typically 100 to 1000× greater (commonly expressed in μg or ng/mL) than in saliva or serum (expressed in pg/mL).^17^ It is important to note that because of these inherent differences in the concentration of melatonin in saliva, serum and urine, it is difficult to make direct comparisons of absolute melatonin in these biological fluids.

**FIGURE 3:**
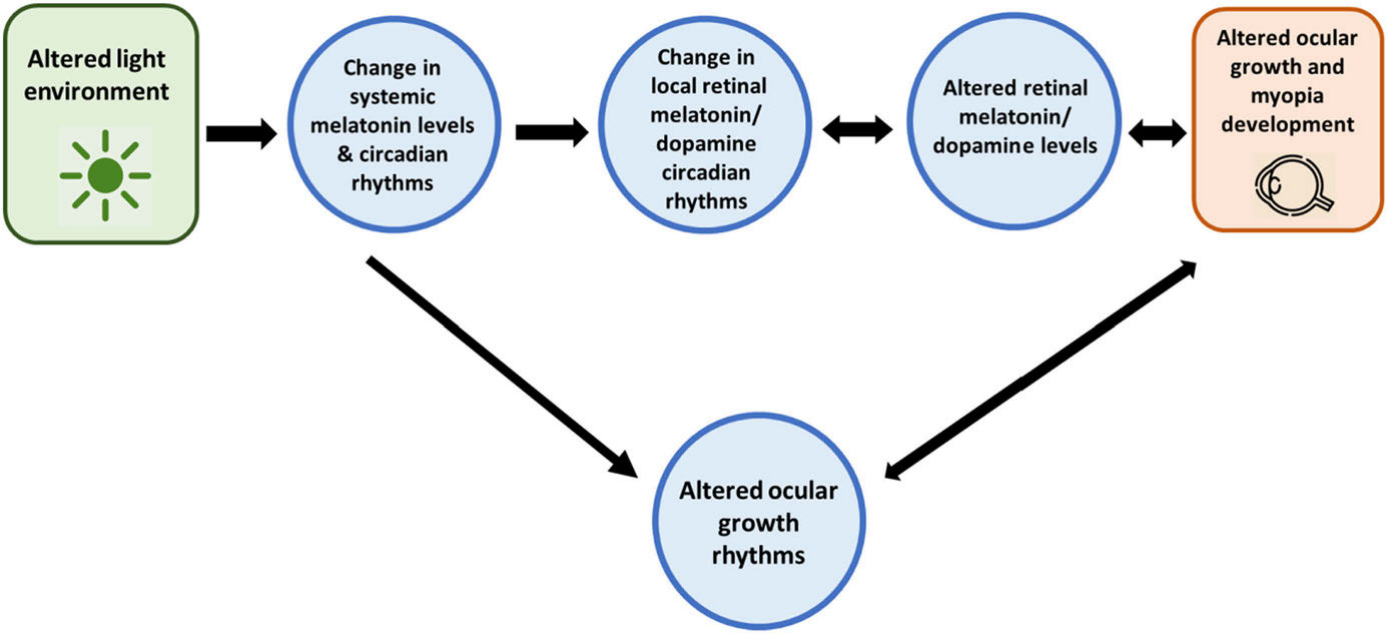
A hypothetical framework that incorporates light exposure, melatonin levels and circadian rhythms, local melatonin and dopaminergic alterations and eye growth rhythms in the development of myopia. Modified from Chakraborty et al.^38^

The exact mechanism through which altered melatonin concentrations may be involved in refractive error development is currently unclear. One possibility is that disruptions in melatonin secretion alter the eye's natural diurnal (or circadian) rhythms, leading to myopia (Figure 3).^67,68^ Melatonin is synthesised not only in the pineal gland but also in the retina^69,70^ under the influence of a circadian clock.^71^ Two mammalian melatonin receptors, MT1 and MT2, have been identified and shown to be present in the eye.^71–73^ The changes in systemic circadian rhythms could potentially affect the local dopamine^74^ and melatonin^69,70^ concentrations and their endogenous rhythms in the retina, which are essential for various structural, metabolic and neurochemical processes, as well as gross functioning of the retina and the eye.^71,73,75^ Disruptions in these local melatonin and dopamine rhythms and/or concentration may also lead to changes in ocular growth and refractive development.^75^ Finally, it has been demonstrated in chicks that systemic administration of melatonin can diffuse into the eye through ocular vasculature and induce choroidal thinning and axial elongation.^76^

The current study has some limitations. First, all included studies were cross-sectional cohort investigations. Future longitudinal studies are warranted to investigate the association between melatonin concentration and diurnal rhythm, as well as the onset and progression of myopia in children. Second, the small number of studies included in this review employed very different methods (single morning blood or saliva sample; 24 h diurnal variation; DLMO and urinary aMT6s) and techniques (LC–MS/MS; double antibody RIA; ELISA) to quantify melatonin concentration, which likely contributed to the inconsistent findings. Newer technologies to monitor core body temperature rhythms using ingestible core temperature capsules (such as e-Celsius Performance, Body Cap, bodycap.us/e-celsius-performance/) may also provide other promising alternatives to measure circadian rhythms in association with refractive error.^77^ Last, these studies were all conducted in different geographical locations and populations, which may also have influenced the observed melatonin profiles and diurnal rhythms.^78^

In conclusion, few studies have investigated the potential role of melatonin circadian dysregulation in myopia. Based on the limited number of studies at this stage, the role of melatonin in myopia development remains unclear. Although there is some evidence of altered melatonin rhythm in myopia, this was not observed consistently. Future large cohort and longitudinal studies with robust methodological approaches (e.g., DLMO with standard sampling rates and lighting conditions) are required to ascertain the causal relationship between melatonin dysregulation and myopia development in humans.

## Supplementary Information

Supplementary file (DOCX 15.5 KB)
