# Supplementary material for: Associations between systemic melatonin and human myopia: A systematic review
Source: Ophthalmic Physiol Opt. 2023 Aug 11;43(6):1478–90. doi: 10.1111/opo.13214 (PMC13400686; doi:10.1111/opo.13214)
Supplement: Supplementary file 1 — Supplementary file (DOCX 15.5 KB) [file 44402_2023_4306019_MOESM1_ESM.docx]

**Appendix 1**

1. **PubMed search strategy**

Search date: 20-01-2023

"Full text, Humans, English, from 2010 - 2022" "((""myopia""[MeSH Terms] OR ""myopia""[All Fields] OR ""myopias""[All Fields] OR (""myope""[All Fields] OR ""myopes""[All Fields]) OR (""myopia""[MeSH Terms] OR ""myopia""[All Fields] OR ""myopic""[All Fields] OR ""myopics""[All Fields]) OR (""myopia""[MeSH Terms] OR ""myopia""[All Fields] OR (""short""[All Fields] AND ""sightedness""[All Fields]) OR ""short sightedness""[All Fields]) OR (""myopia""[MeSH Terms] OR ""myopia""[All Fields] OR (""near""[All Fields] AND ""sightedness""[All Fields]) OR ""near sightedness""[All Fields]) OR (""refractive errors""[MeSH Terms] OR (""refractive""[All Fields] AND ""errors""[All Fields]) OR ""refractive errors""[All Fields] OR (""refractive""[All Fields] AND ""error""[All Fields]) OR ""refractive error""[All Fields])) AND (""loattrfull text""[Filter] AND ""humans""[MeSH Terms] AND 2010/01/01:2022/12/31[Date - Publication] AND ""english""[Language]) AND ((""melatonin""[MeSH Terms] OR ""melatonin""[All Fields] OR ""melatonin s""[All Fields] OR ""melatonine""[All Fields] OR ""melatonins""[All Fields] OR ((""melatonin""[MeSH Terms] OR ""melatonin""[All Fields] OR ""melatonin s""[All Fields] OR ""melatonine""[All Fields] OR ""melatonins""[All Fields]) AND (""rhythm""[All Fields] OR ""rhythm s""[All Fields] OR ""rhythms""[All Fields])) OR ((""melatonin""[MeSH Terms] OR ""melatonin""[All Fields] OR ""melatonin s""[All Fields] OR ""melatonine""[All Fields] OR ""melatonins""[All Fields]) AND (""circadian rhythm""[MeSH Terms] OR (""circadian""[All Fields] AND ""rhythm""[All Fields]) OR ""circadian rhythm""[All Fields]))) AND (""loattrfull text""[Filter] AND ""humans""[MeSH Terms] AND 2010/01/01:2022/12/31[Date - Publication] AND ""english""[Language]))) AND ((fft[Filter]) AND (humans[Filter]) AND (2010:2022[pdat]) AND (english[Filter]))"

1. **EMBASE search strategy**

Search date: 23-01-2023

| #5 | #3 AND (2010:py OR 2011:py OR 2012:py OR 2013:py OR 2014:py OR 2015:py  OR 2016:py OR 2017:py OR 2018:py OR 2019:py OR 2020:py OR 2021:py OR 2022:py)  AND 'human'/de AND 'Article'/it AND 'myopia'/dm |
| --- | --- |
| #4 | #3 AND (2010:py OR 2011:py OR 2012:py OR 2013:py OR 2014:py OR 2015:py OR 2016:py OR 2017:py OR 2018:py OR 2019:py OR 2020:py OR 2021:py OR 2022:py)  AND 'human'/de AND 'Article'/it |
| #3 | ((('myopia'/exp OR myopia OR myopic OR myopes OR 'short sightedness'/exp OR  'short sightedness' OR 'near sightedness'/exp OR 'near sightedness' OR refractive)  AND ('error'/exp OR error) AND melatonin OR melatonin)  AND circadian AND rhythm OR melatonin) AND rhythm |
| #2 | ((('myopia'/exp OR myopia OR myopic OR myopes OR 'short sightedness'/exp OR  'short sightedness' OR 'near sightedness'/exp OR 'near sightedness' OR refractive)  AND ('error'/exp OR error) AND melatonin OR melatonin) AND  circadian AND rhythm OR melatonin) AND rhythm |
| #1 | ('myopia'/exp OR myopia OR myopic OR myopes OR 'short sightedness'/exp  OR 'short sightedness' OR 'near sightedness'/exp OR 'near sightedness'  OR refractive) AND ('error'/exp OR error) |

1. **Web of Science search strategy**

Search date: 23-01-2023

[myopia OR nearsightness OR short-sightedness OR myopes OR myopic OR refractive error (All Fields) and melatonin OR melatonin circadian rhythm OR melatonin rhythm (All Fields) and 2022 or 2021 or 2020 or 2019 or 2018 or 2017 or 2016 or 2015 or 2014 or 2013 or 2012 or 2011 or 2010 (Publication Years) and English (Languages) | 50 results](https://www-webofscience-com.ezproxy-f.deakin.edu.au/wos/woscc/summary/01f472a1-0cf1-4a3c-a247-8b347111b264-6bbba8c3/relevance/1)

1. **Scopus search strategy**

Search date: 23-01-2023

(TITLE-ABS-KEY (myopia OR myopic OR myopes OR short-sightedness OR near-sightedness OR refractive error) AND TITLE-ABS-KEY (melatonin OR melatonin circadian rhythm OR melatonin

rhythm))

1. **Proquest central search strategy**

Search date: 23-01-2023

(myopia OR myopic OR myopes OR short-sightedness OR near-sightedness OR refractive error) AND(melatonin OR melatonin circadian rhythm OR melatonin rhythm) AND la.exact("English") AND(at.exact("Article") AND la.exact("ENG") AND stype.exact("Scholarly

Journals")) AND pd(20100101-20221231)

1. **Ovid search strategy**

Search date: 20-01-2023

((Myopia or myopes or myopic or near sightedness or short sightedness or refractive error) and (Melatonin or melatonin circadian rhythms or melatonin rhythms) and melatonin receptors).af.

1. **LILACS search strategy**

Search date: 20-01-2023

(myopia OR myopes OR myopic OR short-sightedness OR near sightedness OR refractive error) AND (Melatonin OR melatonin circadian rhythm OR melatonin rhythm)

1. **Cochrane search strategy**

Search date: 20-01-2023

"short-sightedness" OR "myopia" OR "near sightedness" OR "myopic" OR "refractive error" in Title Abstract Keyword AND melatonin OR melatonin circadian rhythm OR melatonin rhythm in Title Abstract Keyword AND melatonin receptors in Title Abstract Keyword - with Cochrane Library publication date Between Jan 2010 and Dec 2022 (Word variations have been searched)
